# Systemic Sclerosis-Related Interstitial Lung Disease Amid Latent Tuberculosis and Recurrent Infections

**DOI:** 10.7759/cureus.87826

**Published:** 2025-07-13

**Authors:** Ni Ni Lwin, Wut Y Hlaing, Aprilee Sta Lucia, Ruhina Tasmin

**Affiliations:** 1 Internal Medicine, Harlem Hospital Center, New York, USA

**Keywords:** antifibrotic agents, ild interstitial lung disease, immunosuppressive therapy, multi-professional collaboration, pulmonary fibrosis, ssc-ild, systemic sclerosis (ssc)

## Abstract

Systemic sclerosis is one of the rare autoimmune disorders characterized by fibrosis, damage to the vascular system, and immune dysregulation. Interstitial lung disease is one of the leading causes of morbidity and mortality among pulmonary manifestations of systemic sclerosis. Diagnosing systemic sclerosis-related interstitial lung disease (SSc-ILD) is often delayed due to symptoms such as cough and dyspnea that mimic common respiratory conditions such as pneumonia or bronchitis. We report a 48-year-old woman who presented with recurrent pneumonia-like symptoms. Based on serological and imaging findings, she was diagnosed with SSc-ILD. Her condition improved with antibiotics, immunosuppressants, and antifibrotic agents. The diagnosis and management of SSc-ILD are still challenging, especially when initial symptoms are similar to those of more common conditions such as recurrent pneumonia. An exact diagnosis can only be made by using clinical judgment along with imaging and serologic testing, as shown in this case. Starting treatment early with immunosuppressive and antifibrotic therapies can help slow disease progression and improve quality of life. In complex cases like this, it can be particularly beneficial to have specialists from different fields collaborate, which can make a significant difference in patient care.

## Introduction

Systemic sclerosis, also known as scleroderma, is a chronic, rare autoimmune disease that causes progressive fibrosis of the skin and internal organs, along with microvascular dysfunction and immune dysregulation [1,2]. Systemic sclerosis is typically categorized into two clinical subsets: limited cutaneous systemic sclerosis and diffuse cutaneous systemic sclerosis, both of which may affect the lungs [3]. Among the pulmonary complications associated with systemic sclerosis, interstitial lung disease is the most common and deadliest manifestation, accounting for up to 35% of systemic sclerosis-related mortality [1,4].

The pathogenesis of systemic sclerosis-related interstitial lung disease (SSc-ILD) is complex and not fully understood. However, it involves immune-mediated inflammation, vascular injury, and fibroblast activation, ultimately resulting in pulmonary fibrosis [1,2]. Studies have shown that SSc-ILD does not share the same genetic risk, and the presence of anti-Scl-70 antibodies and the absence of anticentromere antibodies increase the likelihood of progressive interstitial lung disease [5]. Serum Krebs von den Lungen-6 (KL-6) and interleukin-18 (IL-18) correlate positively with the severity of the disease [6]. The clinical presentation can be nonspecific, often mimicking recurrent infections or other chronic lung conditions. Early diagnosis of SSc-ILD using high-resolution CT (HRCT) and serological markers is crucial in optimizing outcomes, including preserving lung function and enhancing quality of life [7]. Here, we present a case of a patient with SSc-ILD who presented with symptoms consistent with recurrent pneumonia. We discuss the challenges of making a correct diagnosis and the significance of a multidisciplinary team's role in the patient's treatment.

## Case presentation

**Figure 1:**
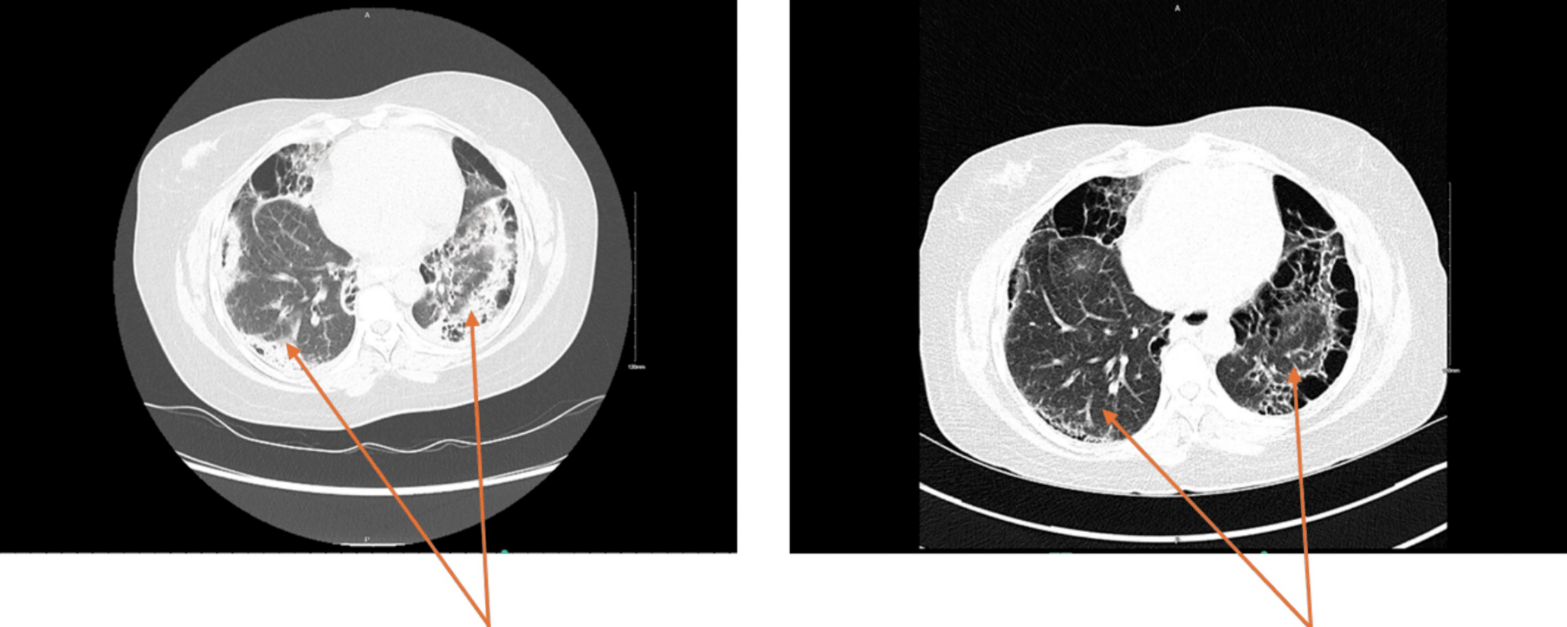
Comparison of High-Resolution Chest CT Scans in a Patient With Systemic Sclerosis-Related Interstitial Lung Disease (SSc-ILD) Before and After Treatment Left: Baseline chest CT showing extensive bilateral lower lobe reticulation, honeycombing, and ground-glass opacities, suggestive of active fibrosing interstitial lung disease. Right: Follow-up CT after three months of immunosuppressive and antifibrotic therapy demonstrating interval improvement in ground-glass opacities and stabilization of fibrotic changes, indicating a partial response to treatment.

A 48-year-old Senegalese woman with a known history of diabetes who works as a secretary has had an intermittent productive cough, exertional dyspnea, weight loss, and anorexia for over a year. She denied any smoking history and reported no family history of autoimmune disease. She has no known history of exposure to environmental or occupational irritants. She had visited the emergency department twice for these symptoms and received empirical antibiotics for presumed bacterial pneumonia. Antibiotics temporarily alleviated her symptoms, but the symptoms recurred within weeks. She tested positive for the QuantiFERON test, prompting a chest X-ray that revealed patchy and reticular opacities. A chest CT scan showed significant peripheral honeycombing with a predominance in the lower lung zones and mid-to-upper lung opacities. These imaging features raised concern for fibrosing interstitial lung disease (Figure 1).

Further tests revealed a high level of antinuclear antibody (ANA) titer at 1:1280 and a strongly positive anti-topoisomerase I antibody (also known as anti-Scl-70), with a value exceeding 8.0, indicating a possible autoimmune connective tissue disease.

Given the CT findings and serological tests, she was diagnosed with SSc-ILD, likely triggered by bacterial pneumonia. Acid-fast bacilli cultures ruled out nontuberculous mycobacterial infection, and an echocardiogram showed no evidence of pulmonary hypertension. She completed a seven-day antibiotic course for bacterial pneumonia. She was started on nintedanib (an antifibrotic medication) and mycophenolate (an immunosuppressive therapy) for interstitial lung disease, along with rifampin for latent tuberculosis. She demonstrated clinical improvement and was discharged with a follow-up plan in pulmonary and rheumatology clinics to monitor disease progression through respiratory symptoms, imaging, and pulmonary function testing (PFT).

## Discussion

This case highlights the diagnostic and management challenges of interstitial lung disease in scleroderma patients. SSc-ILD was diagnosed by a positive ANA, a high anti-topoisomerase I antibody titer, and severe cystic fibrotic changes with honeycombing on HRCT, which also followed expert consensus-based clinical algorithms [1,7,8]. A simple staging system for systemic SSc-ILD classifies disease as limited or extensive, based on simplified HRCT evaluation and forced vital capacity (FVC) estimation, provides more powerful prognostic information than either component in isolation [7]. The treatment of interstitial lung disease in scleroderma patients proves difficult because of the diverse nature of the disease and individual responses to treatment [1,9]. According to the American Thoracic Society (ATS) guidelines, the immunosuppressive medication mycophenolate mofetil and the antifibrotic medication nintedanib are part of the treatment plan [4,10,11]. The benefits of early and targeted therapeutic intervention include stable lung function, symptom alleviation, and radiologic improvement. The accompanying figure illustrates the results before and after three months of antifibrotic and immunosuppressive therapy.

It is essential to continually monitor for the risk of disease progression, as well as treatment-related side effects, including infection. The management of interstitial lung disease associated with scleroderma requires early diagnosis, individualized treatment plans, and collaboration with radiology, pulmonology, and rheumatology specialists [1,9]. New research and continued patient monitoring are also contributing to improved outcomes and informing future therapies.

## Conclusions

SSc-ILD presents both diagnostic and therapeutic challenges, particularly when presenting with nonspecific respiratory symptoms such as recurrent pneumonia. This case highlights the significance of integrating clinical suspicion with serologic and imaging findings to enable timely diagnosis. Stabilizing the disease and enhancing quality of life can be achieved through early intervention with immunosuppressants and antifibrotic therapy. Optimal care mandates a multidisciplinary approach involving infectious disease experts, rheumatologists, and pulmonologists.
